# Molecular and Biochemical Mechanisms of Scutellum Color Variation in *Bactrocera dorsalis* Adults (Diptera: Tephritidae)

**DOI:** 10.3390/insects16010076

**Published:** 2025-01-14

**Authors:** Guangli Wang, Weijun Li, Jiazhan Wu, Ye Xu, Zhaohuan Xu, Qingxiu Xie, Yugui Ge, Haiyan Yang, Xiaozhen Li

**Affiliations:** Department of Plant Protection, College of Agronomy, Jiangxi Agricultural University, Nanchang 330045, China; wgl_2002@126.com (G.W.); liweijun201608@163.com (W.L.); wjzsh1994@163.com (J.W.); hzzhaohuan@163.com (Z.X.); 13803574635@163.com (Q.X.); ygge244@stu.jxau.edu.cn (Y.G.); yhy20055@163.com (H.Y.)

**Keywords:** *Bactrocera dorsalis*, scutellum color variation, single-nucleotide polymorphism, gene function, β-carotene

## Abstract

*Bactrocera dorsalis* (Hendel) is an invasive fruit and vegetable pest, infesting citrus, mango, carambola, etc. We observed that the posterior thoracic scutella of some *B. dorsalis* adults are yellow, some light yellow, and some white in China. Compared with the *B. dorsalis* races with a yellow scutellum (YS) and white scutellum (WS), the race with a light-yellow scutellum (LYS) is dominant in citrus and carambola orchards. To reveal genetic correlates among the three races, the genomes of 22 samples (8 with YS, 7 with LYS, and 7 with WS) were sequenced by high-throughput sequencing technology. Single-nucleotide polymorphism (SNP) annotation showed that there were 17,580 non-synonymous mutation sites located in the exonic region. Principal component analysis based on independent SNP data revealed that the SNPs with LYS were more similar to that with YS when compared with WS. Most genes associated with scutellum color variation were involved in three pathways: oxidative phosphorylation, porphyrin and chlorophyll metabolism, and terpenoid backbone biosynthesis. By comparing the sequences among the three races, we screened out 276 differential genes (DGs) in YS vs. WS, 185 DGs in LYS vs. WS, and 104 DGs in YS vs. LYS. Most genes determining color variation in *B. dorsalis* scutella were located on chromosomes 2–5. Biochemical analysis showed that β-carotene content in YS and LYS was significantly higher than that in WS at any stage of adult days 1, 10, and 20. No significant differences were observed in cytochrome P450 or melanin content in YS, LYS, or WS. Our study provides results on aspects of scutellum color variation in *B. dorsalis* adults, providing molecular and physiological information for revealing the adaptation and evolution of the *B. dorsalis* population.

## Simple Summary

*Bactrocera dorsalis* (Hendel), known as oriental fruit fly, is a global highly invasive insect species. We found that the posterior thoracic scutella of some *B. dorsalis* adults are yellow, some light yellow, and some white in the citrus orchards of China. In this study, we explored the molecular and biochemical mechanisms of scutellum color variation in *B. dorsalis* adults. KEGG enrichment analysis showed that genes associated with scutellum color variation were mainly enriched in these pathways of oxidative phosphorylation, porphyrin and chlorophyll metabolism, and terpenoid backbone biosynthesis. Biochemical analysis showed that β-carotene was one of the main pigments causing the *B. dorsalis* scutella to appear yellow.

## 1. Introduction

Color variation exists extensively in insects and manifests in different color characteristics in different races of a species [1]. For example, *Episyrphus balteatus* adults present two basic color forms: dark and light. The dark form usually occurs in autumn and early spring, while the light form emerges in late spring and summer [2]. There are up to 200 types of color spots in the elytra of *Harmonia axyridis* composed of black or yellow as the base color embedded with yellow or black dot-shaped spots [3,4]. Color in the abdomen of *Eristalis tenax* adults varies greatly but is mostly light-colored, with a few dark-colored [5]. Color variation in insects is an intriguing biological phenomenon, attracting entomologists to explore its ecological, physiological, and genetic mechanisms [6].

The development of sequencing technology has contributed to in-depth research on genetic mechanisms underlying color variation in insects [7]. We retrieved a few studies [4,8] detailing genetic information underlying color variation in different races of an insect species. Lozier et al. (2016) revealed >90,000 and >25,000 single-nucleotide polymorphisms in two geographically disparate color groups of *Bombus bifarius* [6]. Ando et al. (2018) demonstrated repeated inversions within the pannier intron drive diversification of intraspecific color patterns in the elytra of *H. axyridis* [9]. Most research on the genetic mechanism of color variation within a species has focused on plants such as *Brassica napus* [10], *Aquilegia oxysepala* [11], *Pisum sativum* [12], and *Triplophysa siluroides* [13], etc. Color variations in a species are a response to changes in environmental or genetic signals, leading to alterations in internal signaling pathways and the expression of specific genes [14]. Studying the genetic differences associated with color variation in an insect species is essential for revealing its adaptation and evolution under natural environmental conditions.

Pigments enrich insect cuticles, causing insects to exhibit specific color features [15]. Pigments in the insect cuticle are divided into two types. One is the pigments synthesized by insects themselves, comprising melanin, pterin, ommochrome, bilin, papiliochrome, anthraquinone, and aphin, while the other consists of those synthesized by plants and obtained through insect feeding, including carotenoid and flavonoid [16,17]. Among these, melanin exists in almost all insect species [18], such as *Danaus plexippus* [19], *Bactrocera correcta* [20], and *Alphitobius diaperinus* [21]. Cytochrome P450 is a complex family of heme-containing enzymes widely present in all aerobic organisms, including insects, and is involved in the synthesis and metabolism of various pigments [22,23]. Carotenoid, a fat-soluble pigment synthesized by plants, is easily accumulated in insect cuticles after being absorbed by the insect intestine, which causes insects to appear yellow, orange, or red [24]. Davis (2014) reported that color variation is always accompanied by changes in pigment types and concentrations within the insect cuticle [25].

Color variation is an important manifestation of insect biodiversity in specific habitats. The color patterns formed have various biological functions, including aiding in camouflage [26], acting as warning signals to predators [27], attracting mates [28], and helping with thermal regulation [29]. Ellers and Boggs (2010) confirmed that wing melanization is an important determinant of female mating success in the butterfly *Colias philodice eriphyle* [30].

*Bactrocera dorsalis* (Hendel), known as oriental fruit fly, is a highly invasive insect species [31]. It was first recorded in Taiwan, China and the Ryukyu Islands, Japan in 1912 [32], and then invaded Australia, India, Indonesia, Pakistan, the Philippines, Thailand, the United States, and Vietnam [33,34]. In China, the species has spread to most provinces to the south of the Yangtze River, including Anhui, Chongqing, Fujian, Guangdong, Guangxi, Guizhou, Hubei, Hunan, Jiangsu, Jiangxi, Shanghai, Sichuan, Yunnan, and Zhejiang [35]. After over 100 years of adaptation and evolution in different habitats, *B. dorsalis* has developed into a species complex consisting of 85 similar-looking species [36,37,38]. In the species complex, *B. dorsalis* has a broad range of color variation in the terga of the thorax and abdomen, from entirely black to extensively or almost entirely pale [38,39,40]. We, in the citrus orchard in Fuzhou, China, observed that the posterior thoracic scutellum of *B. dorsalis* adults exhibits three different colors, namely yellow, light yellow and white (Figure S1). Initially, they were thought to be three different species in the *Bactrocera* genus. Based on the morphological character of *B. dorsalis* adults (Costal band: confluent with R_2+3_ and remaining narrow and of uniform width to the apex of the wing, occasionally with a slight swelling around the apex of R_4+5_. Abdominal terga III-V: with a medial longitudinal band forming a “T-shaped” pattern with the transverse band at base of tergum III, anterolateral corners of tergum IV small and triangular) [36], and its hosts, damages and responses toward methyl eugenol (A phenylpropanoid compound that is highly attractive to males of *B. dorsalis*) of the three races [41], we considered that the three *B. dorsalis* races with yellow scutellum (YS), light-yellow scutellum (LYS) and white scutellum (WS) belong to a species in *B. dorsalis* complex. Although studies on the genetic diversity of *B. dorsalis* abound [38,42], very little is known about the genetics and physiology of scutellum color variation among the three *B. dorsalis* races.

The objective of our research was to distinguish some differences in the occurrence and distribution of the three *B. dorsalis* races with YS, LYS and WS, and then to explore the molecular and physiological correlates of scutellum color variation. Our study confirmed that the race with LYS was dominant in the three orchards of south China. Compared with the race with WS, the two races with YS and LYS were closer in genetic relationship. Most genes associated with scutellum color variation were involved in the three pathways: oxidative phosphorylation, porphyrin and chlorophyll metabolism and terpenoid backbone biosynthesis. Β-carotene was one of the main pigments causing the *B. dorsalis* scutella to appear yellow.

## 2. Materials and Methods

### 2.1. Adult Trapping

*B. dorsalis* adults were trapped by yellow glue boards (38.5 × 21.5 cm) with glue on both sides (Hefeng, China) in two citrus orchards, Fuzhou (26°21′ N, 119°08′ E) and Nanchang (28°76′ N, 115°85′ E), and in a carambola orchard, Haikou (19°93′ N, 110°35′ E), China. The three regions span approximately 500 km from east to west and 800 km from south to north. It has been confirmed that *B. dorsalis* was distributed in the three regions [43,44]. A total of 68 yellow glue boards (24 in Fuzhou, 22 in Nanchang and 22 in Haikou) were used to capture *B. dorsalis* adults. Yellow glue boards were about 6 m apart from each other. The trapping period in Fuzhou was from 15 to 19 November 2020, and that in Nanchang was from 8 to 15 September 2021, and in Haikou from 16 to 21 July 2021. Captured *B. dorsalis* adults were classified and counted according to the color (yellow, light yellow and white) of scutella.

### 2.2. Sample Preparation

The laboratory population of *B. dorsalis* was derived from 100–150 infested citrus fruits collected in the citrus orchards of Fuzhou in mid-November 2020. The three *B. dorsalis* races with YS, LYS and WS were separated and then were maintained in insect-rearing boxes (78 × 50 × 55 cm) at 27 ± 2 °C, 70 ± 5% RH and 13:11 h (Light: Dark) in the laboratory of entomology, Jiangxi agricultural university. Larvae were fed with an artificial diet containing beer yeast, wheat bran, white sugar, methyl-p-hydroxybenzoate, potassium sorbate and water as the main ingredients. Adults were supplied with yeast powder, diluted honey (1%) and water as food [43].

Adults cultured for 2 days after eclosion were used for genome sequencing. Eight samples were prepared for each *B. dorsalis* race with YS, LYS and WS, containing 10 adults per sample. The eight samples for the *B. dorsalis* race with yellow scutellum (YS) were denoted as YS1, YS2, …, YS8 in sequence, those with light-yellow scutellum (LYS) were denoted as LYS1, LYS2, …, LYS8, and those with white scutellum (WS) were denoted as WS1, WS2, …, WS8. The 24 samples were snap-frozen in liquid nitrogen and then stored at −80 °C prior to DNA extraction.

### 2.3. Genome Sequencing

The 24 samples mentioned above were homogenized under liquid nitrogen in a 2 mL microtube. DNA of these samples was separately extracted using a DNA Rapid Extraction Kit (Shanghai Yubo, Shanghai, China). The samples LYS2 and WS4 were discarded due to contamination during the process of DNA extraction. DNA-seq libraries with approximately 350 bp fragments generated by Covaris DNA ultrasonic breaker were constructed using NEB Next^®^ Ultra™ II DNA Library Prep Kit (Illumina, San Diego, CA, USA) following the manufacturer’s instructions. After passing the quality inspection, the remaining 22 samples were sequenced independently under the Illumina NovaseqTM platform.

The quality of raw sequencing data was controlled using the Benchmarking Universal Single-Copy Orthologs (BUSCO) method under the default setting [45]. After removing the sequencing adapter, low-quality bases and unmeasured bases, clean data were generated using in-house Perl scripts.

### 2.4. SNP Detection

Clean data obtained were aligned with the reference genome (401 Mb) of *B. dorsalis* (https://ftp.ncbi.nlm.nih.gov/genomes/all/GCF/000/789/215/GCF_000789215.1_ASM78921v2/GCF_000789215.1_ASM78921v2_genomic.fna.gz, accessed on 15 July 2022) using Burrows-Wheeler Aligner (BWA) (v0.7.17). The alignment results were deduplicated by SAMTOOLS (v1.17) (parameter: rmdup). Based on the localization results of clean reads in the reference genome, mutation detection on single-nucleotide polymorphism (SNP) was performed using GATK (v3.5-0-g36282e4) UnifiedGenotyper. GATK SelectVariants was used to select SNPs and filter out multiple allele SNPs (MULTIALLELIC), and GATK Variant Filtration and PLINK (v1.90b6.21) were used to filter group SNPs.

### 2.5. Principal Component and Selection Sweep Analysis

Principal component analysis (PCA) was performed by GCTA software (v1.94.1) under default parameters. The SNPs at individual i and k positions are represented by [0, 1, 2]. If individual i is homozygous with the reference allele, then = 0; If mixed, then = 1; If individual i is homozygous with no reference allele, then = 2. The exported results were used to create 3D scatter plots among RC1, PC2 and PC3 using the R package.

Selective clearance analysis of SNPs after quality control was performed by Sweep Finder2 (v1.0). Genetic differentiation coefficient (Fst) and nucleotide diversity (π) were calculated using the F_ST_ software (v9.0) of the PoPoolation2 package (fst-sliding.pl) based on every nucleotide position. The Fst values were arranged in order of size, taking the information from the top 5%, and the ratio of π was taken from the top 5% and bottom 5%. The intersection based on the ratio of Fst to π was screened using Vctools software (v0.1.11) as the genome-wide association. The genome-wide association analysis on the scutellum color characteristic of *B. dorsalis* in different races was performed using the TASSEL (v5.0) (https://tassel.bitbucket.Io, accessed on 8 March 2023) software MLM model. Potential candidate SNPs were screened based on their *p*-value and then annotated using ANNOVAR (v2018Apr16).

### 2.6. Gene Annotation and Enrichment Analysis

The significant SNP loci obtained from selective clearance analysis and genome-wide association analysis were mapped to the *B. dorsalis* reference genome (https://ftp.ncbi.nlm.nih.gov/genomes/all/GCF/000/789/215/GCF_000789215.1_ASM78921v2/GCF_000789215.1_ASM78921v2_genomic.gff.gz, accessed on 8 March 2023) [46]. Subsequently, KEGG enrichment analysis (http://www.genome.jp/kegg/) (accessed on 12 March 2023) on the selected genes was performed using ClusterProfiler (v3.11.0) under default settings [47,48]. We also examined the status of those differential genes between each two *B. dorsalis* races of two types, namely upregulated and downregulated.

### 2.7. Determination of β-Carotene, Cytochrome P450 and Melanin

Robust *B. dorsalis* adults with YS, LYS and WS were selected from our rearing boxes and placed in a freezer for 5 min at −20 °C. Scutella were taken off by a blade and then placed in centrifuge tubes and snap-frozen in liquid nitrogen. Nine samples were prepared separately for testing β-carotene, cytochrome P450 and melanin, including yellow, light-yellow and white scutella from 1-day old, 10-day old and 20-day old adults after eclosion, respectively. For each sample, 3 biological replicates were prepared, and each replicate contained 30 scutella.

The content of β-carotene in *B. dorsalis* scutella was determined by high-performance liquid chromatography (HPLC, Agilent 1130, Agilent Technologies Inc., Shanghai, China). The samples mentioned above were weighed accurately with analytical balance (XPR106DUHQ/AC, METTLER TOLEDO, Shanghai, China) and then homogenized under liquid nitrogen in a mortar. A total of 20 mL of a mixture of 80% petroleum ether and 20% acetone (1:1, *v*/*v*) was added, sealed with cling film, and then placed in a water bath at 30 °C for 12 h, extracting β-carotene. After being filtered by the 0.45 μm filter membrane, the extracted solution with β-carotene was evaporated to dry, and 20 mL of mobile phase was added, which was used for chromatographic analysis. Standard samples of β-carotene (Biochemika Fluka, ≥97.0%, Lefu Biotechnology Co., Ltd., Shenzhen, China) were diluted with acetone as the solvent at 50 μg/mL and stored in a refrigerator at 2 °C as a control. The chromatographic detection conditions for β-carotene were as follows: the mobile phase was 60% acetonitrile + 20% ethyl acetate + 20% methanol (1:1:1, *v*/*v*/*v*), the detection wavelength was 450 nm, the column temperature was 25 °C, and the flow rate of mobile phase was 0.8 mL/min.

The content of cytochrome P450 in *B. dorsalis* scutella was assayed spectrophotometrically as described by Li et al. (2017) [49]. Weighed samples were homogenized in 2 mL of 0.1 mol/L phosphate buffer (containing 0.1 mmol/L PMSF, 0.1 mmol/L DTT, 1 mmol/L EDTA, 1 mmol/L PTU and 10% Glycerol) for 2 min at 4 °C. The crude homogenate was centrifuged at 10,000 r/min for 30 min at 4 °C, and then the supernatant was taken as samples for determining the content of cytochrome P450. The supernatant fluid was diluted with phosphate buffer to a protein mass concentration of approximately 1 mg/mL, and then sodium hydrosulfite was added. After standing still for 5 min, the sample was divided into 2 portions and separately poured into the sample cup and reference cup. Pre-prepared CO (obtained by adding concentrated sulfuric acid dropwise to formic acid) was added to the sample cup for 1 min, standing for 10 min. The D value in the range of 400–500 nm was measured using a UV spectrophotometer (Thermo–Fisher Scientific, Guangzhou, China) with multiple scans. Cytochrome P450 content (nmol/mg) = [ΔOD (450–490 nm) × 1000]/[91 × protein mass concentration (mg/mL)].

The content of melanin in different color scutella was also determined by HPLC. Samples were weighed accurately and then broken under liquid nitrogen. A total of 2 mL of 2% papain and 2 mL of 0.1 mol/L phosphate buffer was added, and then the samples were shaken for 24 h at 55 °C. The crude extract was centrifuged at 9500 r/min for 5 min at 25 °C, and then the supernatant was discarded. The sediment was washed with petroleum ether, anhydrous ethanol, and distilled water twice to obtain a sample containing melanin. A total of 2.0 mL of 1 mol/L K_2_CO_3_ and 0.2 mL of 3%H_2_O_2_ was added, and the sample was placed in a water bath for 20 min at 100 °C. After cooling, 0.1 mL of 10% Na_2_SO_3_ was added to terminate the reaction and adjust the pH to 1.0 by 6 mol/L HCl. After centrifugation at 9500 r/min for 5 min, the supernatant was taken and extracted twice with 10 mL anhydrous ether, and the organic phases were combined. Inkfish melanin was used as a control (Sigma/Merck, Shanghai, China), which contained 95% melanin and 5% binding proteins. The HPLC detection conditions were the same as those mentioned above, except for a detection wavelength of 275 nm.

### 2.8. Data Analyses

All statistical analyses were conducted using SPSS 19.0, followed by Tukey’s honestly significant difference (HSD) test (*p* < 0.05). The percentages of three *B. dorsalis* races in three orchards and the content of β-carotene, cytochrome P450 and melanin in three color scutella were expressed as mean ± standard error. Related graphics were drawn using GraphPad Prism 8.0.1.

## 3. Results

### 3.1. Color Variation of B. dorsalis Scutella

**Figure 1:**
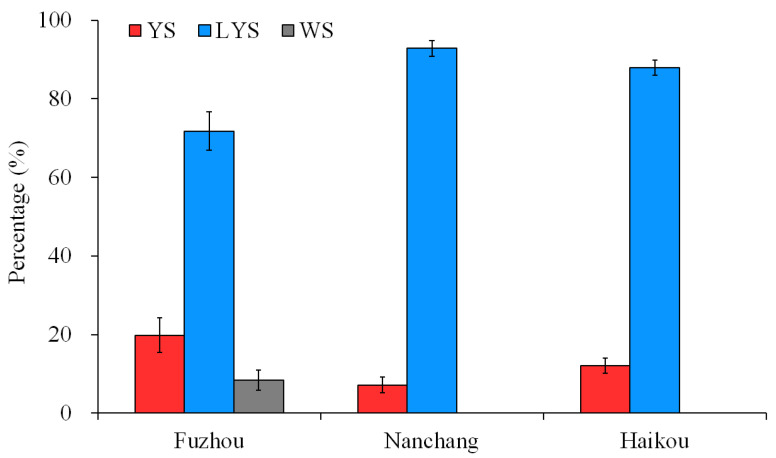
Percentage of the three *B. dorsalis* races with yellow, light-yellow and white scutella. YS, yellow scutellum; LYS, light-yellow scutellum; WS, white scutellum.

The posterior thoracic scutellum of some *B. dorsalis* adults appears yellow, some light yellow, and some white. The estimated chromaticity values of yellow, light-yellow and white scutella are shown in Table S1. Based on the capture by yellow glue board in the citrus and carambola orchards of Fuzhou, Nanchang and Hainan, China, the percentages of the three *B. dorsalis* races with YS, LYS and WS were calculated, respectively. In Fuzhou, a total of 1722 *B. dorsalis* adults were obtained, with 19.83% being YS, 71.80% being LYS, and 8.37% being WS. The number of adults with LYS captured was significantly higher than those with YS and WS (F = 29.515, df = 2, 69, *p* < 0.05). In Nanchang and Hainan, *B. dorsalis* adults with LYS captured accounted for 92.83% and 87.86%, respectively, and no adults with WS were captured (Figure 1). These results suggested that the *B. dorsalis* race with LYS was dominant in the citrus orchards of China at present.

### 3.2. Genome Data of Three B. dorsalis Races

Twenty-two samples of the three *B. dorsalis* races with YS (8), LYS (7) and WS (7) were sequenced under the Illumina Hiseq platform, which generated 14,430,855 raw reads in YS, 14,665,016 in LYS, and 13,896,407 in LYS. After removing connector sequences, low-quality bases and undetected bases, 14,374,050 clean reads in YS, 14,609,030 in LYS and 13,840,739 in WS were retained. The sequencing quality of samples was high (YS: Q20 ≥ 97.88%, Q30 ≥ 93.46%; LYS: Q20 ≥ 97.87%, Q30 ≥ 93.43%; WS: Q20 ≥ 97.68%, Q30 ≥ 92.96%), and the GC content was normal (YS: 37.43–37.96%; LYS: 37.56–38.24%; WS: 37.06–37.89%). The average mapping rate of LYS genomes on the reference genome was 83.65%, the average sequencing depth was 10.26, and the average coverage was 90.73%, and those of WS genomes were 83.14%, 9.94 and 90.49%, respectively (Table 1).

### 3.3. SNP Annotation of Genome Data

**Figure 2:**
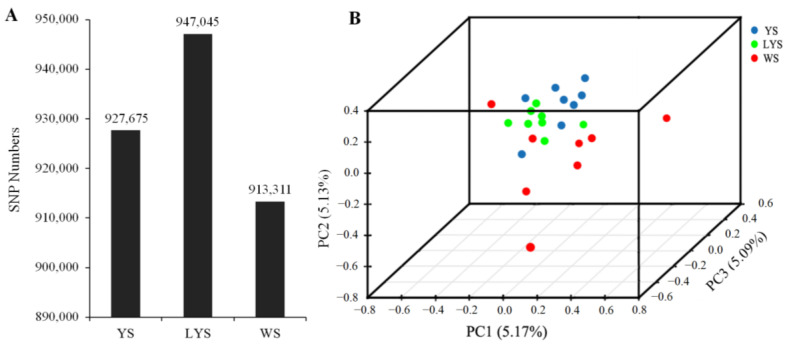
Principal component analysis was generated with independent SNP data from the three *B. dorsalis* races with yellow, light-yellow and white scutellum. (**A**) Column chart of SNP numbers; (**B**) 3D scatter plot of principal component analysis. The dissimilarity of the three *B. dorsalis* races with YS, LYS and WS is based on the unweighted UniFrac metrics. The percent variation explained by each race is shown on the axis.

A total of 1,912,256 SNPs were detected from 22 genome samples, including 1,176,971 SNPs of transition mutation and 735,285 SNPs of transversion mutation. The ratio between transition and transversion (Ts/Tv) was 1.60 (Table S2). We obtained 927,675 SNPs from *B. dorsalis* race with YS, 947,045 SNPs from that with LYS and 913,311 SNPs from that with WS (Figure 2A). The annotation of SNPs showed that there were 84,225 nucleotide mutation sites located in the exonic region, including 17,580 non-synonymous mutation sites and 66,645 synonymous mutation sites (Table S2).

The relationship among the three *B. dorsalis* races with YS, LYS and WS was revealed by principal component analysis based on the independent SNP data present in 22 samples. The result showed that the SNPs of the race with YS and that with LYS were highly overlapping, while the SNPs of the race with WS and the other two races with YS and LYS were relatively separated (Figure 2B), which indicated that there were some gene flows among the three *B. dorsalis* races with YS, LYS and WS, especially between the two races with YS and LYS. 

### 3.4. KEGG Pathway Enrichment Analysis

**Figure 3:**
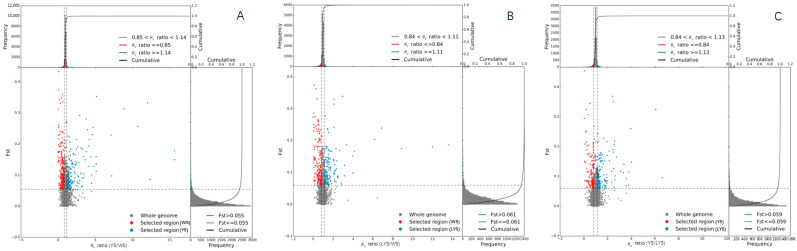
Selection sweep analysis between each two *B. dorsalis* races with YS, LYS and WS. (**A**) Selection sweep analysis of YS vs. WS; (**B**) Selection sweep analysis of LYS vs. WS; (**C**) Selection sweep analysis of YS vs. LYS. The frequency distribution diagram above and the frequency distribution diagram on the right correspond to the π ratio on the abscissa and Fst value on the ordinate, respectively. The dot diagram in the middle represents the corresponding Fst and π ratios in different windows. The top red and blue areas are the top 5% areas selected by π, the green areas are the top 5% areas selected by Fst, and the middle blue and red areas are the intersection of Fst and π, which are the candidate sites.

The quantiles of the genetic differentiation index (Fst) and nucleotide diversity ratio (π ratio) between the two *B. dorsalis* races with YS and WS at 95% level were 0.85 and 1.14, respectively. Therefore, we selected the intersection of Fst ≥ 0.85 and π ratio ≥ 1.14 to perform gene annotation and then performed KEGG enrichment analysis on the candidate genes detected using ClusterProfiler (Figure 3).

**Figure 4:**
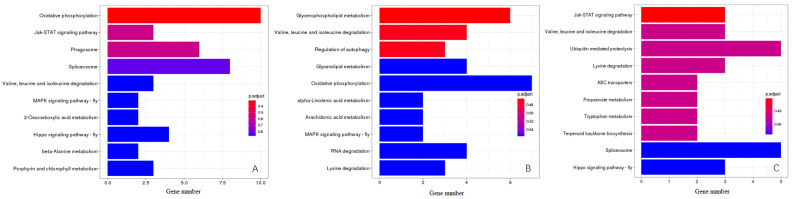
KEGG pathway enrichment analysis of candidate genes between each two *B. dorsalis* races with YS, LYS and WS. (**A**) Enriched pathway of genes between YS vs. WS; (**B**) Enriched pathway of genes between LYS vs. WS; (**C**) Enriched pathway of genes between YS vs. LYS. The *X*-axis represents the number of genes, the *Y*-axis represents the pathway name, and the color represents the significance of enrichment in that pathway.

**Figure 5:**
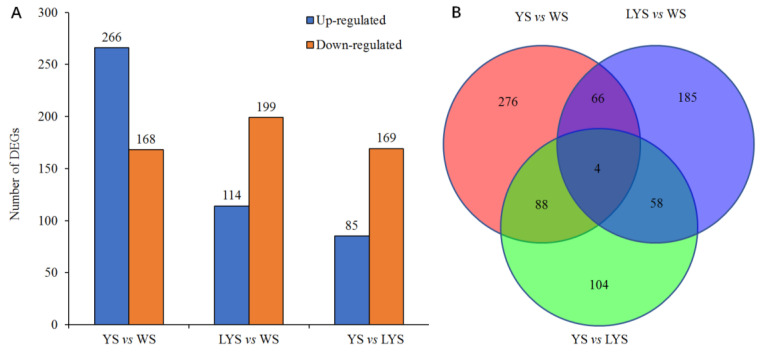
Column chart and Venn diagram of the numbers of differential genes (DGs) between each of two *B. dorsalis* races with YS, LYS and WS. (**A**) The number of upregulated and downregulated DGs; (**B**) The number of overlapped and specific DGs.

The KEGG Orthology (KO) classification showed that 43 genes in YS vs. WS, 37 genes in LYS vs. WS and 30 genes in YS vs. LYS were annotated as being associated with 3 primary pathways as environmental information processing, metabolism and genetic information processing (Figure 4, Table 2). In the category of environmental information processing, 3 genes in YS vs. WS were involved in the Jak-STAT signaling pathway (Figure 4A), and 2 genes in LYS vs. WS were involved in the MAPK signaling pathway (Figure 4B). In the category of metabolism, 3 genes in LYS vs. WS were enriched in porphyrin and chlorophyll metabolism (Figure 4A), and 2 genes in YS vs. LYS were enriched in terpenoid backbone biosynthesis (Figure 4C). In the category of genetic information processing, 8 genes in YS vs. WS (Figure 4A) and 5 genes in YS vs. LYS (Figure 4C) were involved in the Spliceosome pathway. The highest proportion of genes in YS vs. WS (10) and LYS vs. WS (7) was oxidative phosphorylation (Figure 4A,B), while that in YS vs. LYS was ubiquitin-mediated proteolysis (5) and Spliceosome (5). The metabolic pathway accounted for the largest proportion of genes, including Valine, Leucine and Isoleucine degradation, 2-oxocarboxylic acid metabolism, beta-alanime metabolism, Porphyrin and chlorophyll metabolism, Glycerophospholipid metabolism, Glycerolipid metabolism, alpha-Linolenic acid metabolism, Arachidonic acid metabolism, RNA degradation, Lysine degradation, Propanoate metabolism and Tryptophan metabolism (Figure 4).

### 3.5. Differential Genes Between Each Two B. dorsalis Races

We screened out a total of 434 differential genes (DGs) based on the pairwise comparison of genomes between each two *B. dorsalis* races with YS, LYS and WS, which consisted of 266 upregulated DGs and 168 downregulated DGs. Using similar methods, 313 DGs of LYS vs. WS were screened out, of which 114 DGs were upregulated and 199 DGs were downregulated. Only 254 DGs of YS vs. LYS were obtained, containing 85 upregulated DGs and 169 downregulated DGs (Figure 5A). Venn diagram showed that there were 276 specific genes in YS vs. WS, 185 in LYS vs. WS and 104 in YS vs. LYS. There were only 4 overlapped genes among the three *B. dorsalis* races (Figure 5B).

### 3.6. Genome-Wide Association Analysis Between Each Two B. dorsalis Races

We here screened out 21 genes associated with the color variation of *B. dorsalis* scutellum through genome-wide association analysis. Except for gene *Ube4B* on chromosome 1 and *Dhdds* unplaced scaffold, all other genes were located on chromosomes 2–5. Among them, 5 genes *Prp19*, *Fancl*, *Ube4B*, *Nedd4* and *Herc4* were enriched in the pathway of ubiquitin-mediated proteolysis, involving in the regulation of color variation in YS vs. LYS. Three genes, *Acat2*, *LOC105223844* and *Dhdds,* were enriched in the pathway of terpenoid backbone biosynthesis, regulating the color variation in YS vs. LYS, and 2 genes, *blw* and *LOC105229962,* were enriched in the pathway of oxidative phosphorylation, regulating the color variation in YS vs. WS and LYS vs. WS (Table 3). These genes played certain roles separately in the transport, synthesis, and degradation of pigments in the *B. dorsalis* scutellum.

### 3.7. Pigment Contents

#### 3.7.1. β-Carotene

The contents of β-carotene in YS, LYS and WS of *B. dorsalis* were determined by HPLC. In YS and LYS, the β-carotene contents increased over time and showed significant differences among adults on days 1, 10 and 20 after eclosion (YS: F = 126.878, df = 2, 6, *p* < 0.05; LYS: F = 39.188, df = 2, 6, *p* < 0.05). Compared with adult day 1 (newly emerged), the β-carotene contents in YS of adult days 10 and 20 increased by 112.72% and 127.19%, while that in LYS of adult days 10 and 20 increased by 96.85% and 108.44%, respectively. β-carotene in WS of adults on days 1, 10 and 20 had no significant change in content (F = 2.536, df = 2, 6, *p* = 0.159).

**Figure 6:**
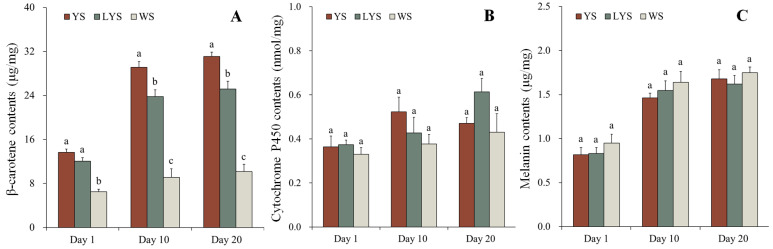
Changes in pigment contents in different color scutella of *B. dorsalis* adult days 1, 10 and 20 after eclosion. At each stage, different letters above the bars indicate significant differences (Tukey’s HSD test, *p* < 0.05). (**A**) β-carotene; (**B**) cytochrome P450; (**C**) melanin.

At each stage, the change in β-carotene content was analyzed in yellow, light-yellow and white scutella of *B. dorsalis*. In adults, day 1 (newly emerged), the β-carotene contents in YS and LYS were 2.11 and 1.86 times higher than that in WS (F = 44.755, df = 2, 6, *p* < 0.05). However, no significant differences were observed between YS and LYS. In adults day 10, the β-carotene contents in YS and LYS were 3.18 and 2.60 times as high as that in WS (F = 62.265, df = 2, 6, *p* < 0.05), while in adults day 20, those in YS and LYS were 3.05 and 2.47 times as high as that in WS (F = 80.790, df = 2, 6, *p* < 0.05) (Figure 6A).

#### 3.7.2. Cytochrome P450

The contents of cytochrome P450 in YS, LYS and WS of *B. dorsalis* adults were detected by UV spectrophotometer. Although the cytochrome P450 contents in scutella of adults days 10 and 20 slightly increased, it was not significantly different compared with that of adults day 1 (newly emerged) (YS: F = 2.624, df = 2, 6, *p* = 0.152; LYS: F = 5.178, df = 2, 6, *p* = 0.049; WS: F = 0.774, df = 2, 6, *p* = 0.502). Similarly, there were also no significant differences in the cytochrome P450 contents among different color scutella at each stage of adults (Day 1: F = 0.420, df = 2, 6, *p* = 0.675; Day 10: F = 1.436, df = 2, 6, *p* = 0.309; Day 20: F = 2.440, df = 2, 6, *p* = 0.168) (Figure 6B). It was clear that the contents of cytochrome P450 were relatively stable, whether in the different color scutella at a certain stage or in the same color scutella at different stages of *B. dorsalis*.

#### 3.7.3. Melanin

We measured the contents of melanin in YS, LYS and WS of *B. dorsalis* adult day 1, 10 and 20 by HPLC. The melanin contents in YS, LYS and WS significantly increased with the prolongation of time (YS: F = 28.921, df = 2, 6, *p* < 0.05; LYS: F = 21.543, df = 2, 6, *p* < 0.05; WS: F = 18.847, df = 2, 6, *p* < 0.05). Compared with newly emerged adults, the melanin contents in YS of adult days 10 and 20 increased by 179.01% and 205.48%, while that in WS of adult days 10 and 20 increased by 172.53% and 184.02%, respectively. However, it had no significant change in the melanin contents among different color scutella at each stage of adults (Day 1: F = 0.760, df = 2, 6, *p* = 0.508; Day 10: F = 0.762, df = 2, 6, *p* = 0.507; Day 20: F = 0.508, df = 2, 6, *p* = 0.626) (Figure 6C).

## 4. Discussion

The different races of *B. dorsalis* exhibit different colors or color patterns in the thorax and abdomen [1,50]. Several studies have studied the color variation among *B. dorsalis* races, which focused on intraspecific variation [42,51] and phylogenetic development [38,52]. Color variation in insects is associated with complex physiological processes and genetic mechanisms [53]. Our study represents the most comprehensive exploration undertaken to date on the molecular and physiological correlates of color variation in the scutellum of *B. dorsalis* adults. The result is helpful for a better understanding of the color variation mechanism of the species in scutella.

### 4.1. Ecological Differences Among the Three B. dorsalis Races

Our research confirmed that the race with LYS was widely distributed in Fuzhou, Nanchang and Haikou, while that with WS was only distributed in Fuzhou. Actually, the races with YS and LYS were common in southern China, mainland Southeast Asia and southern Thailand [54,55], while the race with WS was not common. We noticed literature by Leblanc et al. (2015) [51], recording the scutella of some species in *B. dorsalis* complex collected in Australia, Cambodia, French Polynesia, Hawaii, Laos, Malaysia and Thailand displaying white or grayish-white, similar to that of the race with WS we found in Fuzhou. It has been proved that those species in the *B. dorsalis* complex have a high degree of host specialization [56]. The three races we mentioned here are important economic species that feed on citrus, mango, starfruit, etc. [57], while most of those races recorded by Leblanc et al. (2015) [51] are non-economic species (No record about their hosts). Therefore, we did not consider that the race with WS mentioned here is the same as a species recorded by Leblanc et al. (2015) [51]. However, it is still unclear whether the race with WS comes from individual heteromorphosis or some other regions.

### 4.2. Genetic Relationship Among the Three B. dorsalis Races

More than 13 million clear reads per sample were generated under the Illumina Hiseq platform, which provided sufficient genome data for analyzing non-synonymous mutation sites, differential genes (DGs) and the function of DGs between each two *B. dorsalis* races. The values of Q20 (≥97.68%) and Q30 (≥92.96%) demonstrated that the genome data obtained here were qualified for localizing the non-synonymous mutation points, identifying the functional genes and analyzing the function of DGs [58,59]. However, more than 16% of genes were not annotated on the reference genome of *B. dorsalis* in https://ftp.ncbi.nlm.nih.gov/genomes/all/GCF/000/789/215/GCF_000789215.1_ASM78921v2/GCF_000789215.1_ASM78921v2_genomic.fna.gz (accessed on 8 March 2023) through BWA software (v0.7.17). This may be due to the lack of annotation information on the reference genome for some genes with low expression levels [7].

It is very common that the non-synonymous mutation of SNP sites causes obvious phenotypic variations in insects [60,61]. In the exonic region of DNA, 17,580 non-synonymous mutation sites were found by the SNP functional annotation of the samtools package. The mutation sites relating to the color variation of *B. dorsalis* scutella might be among them. We did not determine which mutations of SNPs were associated with the color variation of *B. dorsalis* scutella. The principal component analysis based on the independent SNP data indicated that there were close genetic connections among the three *B. dorsalis* races, especially between the race with YS and that with LYS. The genetic connections could be reflected in the biological information in the three *B. dorsalis* races in our studying regions. Compared to the *B. dorsalis* race with WS, the two races with YS and LYS have more similar densities and distributions (Figure 1), which indicates a higher probability of hybridization and the exchange of genetic material between them.

### 4.3. Molecular Correlates of Scutellum Color Variation

The expression of DGs causes changes in the phenotype or biology of insects [62]. Generally, the higher the number of DGs, the greater the phenotypic changes in insects [61]. We considered that the specific expression of related DGs determines the color feature of *B. dorsalis* scutella. Our research showed that there were the highest numbers of DGs between the race with YS and that with WS, and they also exhibited the greatest differences in the color feature of scutella, from white to yellow (Figure 4). Similar studies, including those for *Nilaparvata lugens* [63] and *Heliconius erato* [64], have also confirmed that the specific DGs may be expressed differentially in response to body color changes.

KEGG pathway enrichment analysis revealed that the function of DGs among the *B. dorsalis* races with different color scutella was enriched in multiple biochemical pathways, including oxidative phosphorylation, ubiquitin-mediated proteolysis, Valine, Leucine and Isoleucine degradation, terpenoid backbone biosynthesis, glycerophospholipid metabolism and Jak-STAT signaling pathway, etc. (Figure 4). By checking each biochemical pathway, we found that the oxidative phosphorylation pathway (Figure 4A,B), porphyrin and chlorophyll metabolism (Figure 4A), and terpenoid backbone biosynthesis (Figure 4C) were closely related to the color formation of *B. dorsalis* scutella. Among them, the oxidative phosphorylation pathway is an important energy metabolism pathway, which provides energy in the form of ATP for organisms, including insects [65]. The energy generated by this pathway is favorable for synthesizing pigments in insect cuticles, such as quinones [66]. Quinones contain highly conjugated structures, with para quinones mostly appearing yellow and ortho quinones mostly appearing red or orange [67]. Yellow scutellum of *B. dorsalis* adult may contain para quinones. Roth and Stay (1958) [68] confirmed that para quinones occurred in the cuticle of some arthropods such as *Diploptera punctate*. The porphyrin and chlorophyll metabolism also plays an important role in the color formation of *B. dorsalis* scutella, as suggested by the enrichment analysis on the DGs between the two races with YS and WS (Figure 4A). Chlorophyll is the pigment for plant photosynthesis, containing a porphyrin ring composed of four pyrroles [69]. During the process of porphyrin and chlorophyll metabolism, chlorophyll is first hydrolyzed into chlorophyllin acid and then further metabolized into various pigments, such as carotenoids and flavonoids [70]. Carotenoids (β-carotene) have been determined by HPLC in the *B. dorsalis* scutella (Figure 6A). We considered that some carotenoids in scutella come from food such as mature citrus, and others may come from the metabolism of chlorophyll that occurs in *B. dorsalis*. Terpenoids are widely present in insects, such as *Nauphoeta cinerea* and *Periplaneta Americana*, and are an important component of insect cuticles [71,72]. It is also the main component of pigments, including flavonoids appearing yellow and anthocyanins appearing red or blue [73]. We found the terpenoid backbone biosynthesis pathway in the enrichment analysis of the DGs between the two races with YS and LYS (Figure 4), which indicated that flavonoids may also be synthesized in *B. dorsalis* and enriched in their scutella.

Overall, the color of *B. dorsalis* scutellum may be the result of a combination of multiple pigments, including para quinones, carotenoids, flavonoids, etc. Other biochemical pathways may also be involved in the color formation of *B. dorsalis* scutellum, but they are not analyzed here due to the unclear biochemical process at present.

### 4.4. Physiological Correlates of Scutellum Color Variation

There are various types of pigments in insect cuticle. We measured the contents of β-carotene, cytochrome P450 and melanin in YS, LYS and WS at adult days 1, 10 and 20 after eclosion. The three pigments or compounds were chosen because they represent the main types of pigments in insect cuticles, and they are also expected to display specific biochemical processes of their expression or enrichment in the *B. dorsalis* scutellum. Our results showed that there were abundant β-carotene, cytochrome P450 and melanin in *B. dorsalis* scutella, but their contents varied greatly in different color scutella and at different adult stages. Many studies on the type, formation and function of insect pigments were conducted [74,75,76], but little on the content of specific pigments in insect scutella [3].

Our research found that the content of β-carotene is highest in YS, followed by LYS, and lowest in WS, indicating that β-carotene is an important pigment causing scutellum to appear yellow. β-carotene was also detected in the cuticles of other insects, including *Aglais urticae* [77], *Pieris brassicae* [78], *Menduca sexta* [66] and *Drosophila melanogaster* [79], etc. The content of β-carotene in *B. dorsalis* scutella was lower on adult day 1 than on adult days 10 and 20. Previous research confirmed that carotenoids in insect cuticles came from food or the degradation of related compounds such as chlorophyll from food, as no carotenoids were synthesized in insects [17]. Therefore, we suggested that most of the β-carotenes were obtained by feeding at the larval stage and then stored in certain tissues. After the pupal stage, β-carotenes gradually accumulate in the yellow scutella or other related yellow tissues of *B. dorsalis*. The similar biochemical processes involved in the formation of insect body color have been documented in other insects, such as *Heliconius* spp. [80] and *Drosophila melanogaster* [66].

Cytochrome P450 is not a pigment but is involved in the synthesis and metabolism of some pigments, such as porphyrin [22], which affects the color of insect cuticles. Cytochrome P450 was detected in the *B. dorsalis* scutella, and there was no obvious difference in its content among yellow, light-yellow and white scutella. We considered that cytochrome P450 is relatively evenly dispersed in various tissues of *B. dorsalis*, as, in the other insects, it is involved in various physiological activities, such as the synthesis of ecdysteroids [81], the metabolism of insecticides [82] and plant secondary substances [83], which indicated that it was not enriched in a specific tissue of insects. The changes in melanin content were roughly similar to that of cytochrome P450 content in different color scutella and at different adult phases. Unlike with cytochrome P450, the melanin content increased obviously along with the development of *B. dorsalis* adults (Figure 6). Based on the color change in the *B. dorsalis* cuticle, we assumed that more melanin accumulates in other black tissues of the body wall outside the scutella.

Compared with β-carotenes, the content of cytochrome P450 and melanin was lower, and no great changes were observed in their contents in *B. dorsalis* scutella. We demonstrated that β-carotenes played an important role in the yellow presentation of *B. dorsalis* scutella among these pigments or relative compounds. Other pigments undetected here, such as para quinones and flavonoids, may also exist in *B. dorsalis* scutella and affect the color presentation of *B. dorsalis* scutella.

The study provides a clear signal that the *B. dorsalis* race with LYS has a wider range of distribution in China and a stronger ability to adapt. The genes involved in the oxidative phosphorylation pathway, porphyrin and chlorophyll metabolism and terpenoid backbone biosynthesis are associated with the formation of *B. dorsalis* scutellum color. β-carotene is one of the main pigments that cause *B. dorsalis* adult scutellum to exhibit yellow or light yellow. Our results provided valuable clues for understanding the mechanisms associated with the color variation of scutellum in *B. dorsalis* adults.

## Figures and Tables

**Table 1:** Genome data of 22 samples from the three *B. dorsalis* races with YS, LYS and WS.

Sample	Raw Read	Clean Read	Q20 (%)	Q30 (%)	GC (%)	Mapping Rate (%)	Sequencing Depth	Coverage (%)
YS1	16,175,193	16,114,275	98.01	93.77	37.60	82.96	10.92	91.32
YS2	13,674,576	13,624,567	98.05	93.85	37.57	83.49	9.86	90.61
YS3	12,807,459	12,753,577	97.88	93.50	37.84	82.96	9.75	89.58
YS4	14,123,606	14,066,036	97.88	93.46	37.43	83.89	10.05	91.01
YS5	13,479,907	13,422,541	97.98	93.73	37.56	82.62	9.85	90.69
YS6	13,698,693	13,646,031	97.91	93.53	37.58	83.32	9.97	90.41
YS7	15,930,348	15,869,990	98.02	93.81	37.58	83.05	10.66	91.33
YS8	15,557,059	15,495,381	98.01	93.76	37.96	82.84	10.68	90.73
LYS1	14,689,206	14,633,672	97.99	93.70	37.74	83.52	10.30	91.18
LYS3	15,045,434	14,990,061	97.99	93.76	37.70	83.79	10.50	91.02
LYS4	14,472,519	14,422,056	97.99	93.76	37.56	83.64	10.36	90.61
LYS5	14,444,804	14,390,911	98.04	93.85	37.79	83.75	10.28	90.56
LYS6	15,877,274	15,817,763	98.02	93.70	38.24	83.63	11.11	89.86
LYS7	13,789,486	13,735,167	97.87	93.43	37.65	83.23	10.08	90.36
LYS8	14,336,392	14,273,582	98.09	93.97	37.91	83.96	9.20	91.54
WS1	15,320,793	15,258,254	97.77	93.17	37.06	82.41	10.34	91.02
WS2	14,961,581	14,896,256	97.73	92.96	37.06	81.71	9.52	90.33
WS3	15,680,005	15,620,251	97.80	93.17	37.06	83.44	10.68	91.42
WS5	12,445,398	12,394,219	97.82	93.28	37.26	83.52	9.62	90.04
WS6	14,995,683	14,944,984	98.09	93.91	37.89	83.81	10.57	90.75
WS7	12,230,812	12,182,772	97.93	93.61	37.33	83.99	9.61	89.69
WS8	11,640,577	11,588,437	97.68	93.02	37.35	83.13	9.22	90.16

Samples LYS2 and WS4 were discarded due to contamination.

**Table 2:** Main enrichment pathway classification and gene numbers enriched in specific pathways.

First Level Classification	Secondary Classification	Ko Classification	Ko Pathway	Paired Samples	Gene Numbers
Environmental information processing	Signal transduction	ko04630	Jak-STAT signaling pathway	YS vs. WS	3
				LYS vs. WS	-
				YS vs. LYS	3
		ko04013	MAPK signaling pathway–fly	YS vs. WS	2
				LYS vs. WS	2
				YS vs. LYS	-
		ko04391	Hippo signaling pathway–fly	YS vs. WS	4
				LYS vs. WS	-
				YS vs. LYS	3
Metabolism	Amino acid metabolism	ko00280	Val, Leu and Ile degradation	YS vs. WS	3
				LYS vs. WS	-
				YS vs. LYS	3
	Other amino acid metabolism	ko00410	Beta-Alanime metabolism	YS vs. WS	2
				LYS vs. WS	-
				YS vs. LYS	1
Genetic information processing	Translation	ko03040	Spliceosome	YS vs. WS	8
				LYS vs. WS	7
				YS vs. LYS	5

**Table 3:** Candidate genes associated with the color variation of *B. dorsalis* scutellum.

Gene Name	Gene ID	Chromosome	Position in Chromosome	Ko Pathway	Scutellum Color
*blw*	105229678	3	NC_064305.1 (87342785–87345945)	Oxidative phosphorylation	YS vs. WS LYS vs. WS
*LOC105229962*	105229962	3	NC_064305.1 (79775155–79777217)	Oxidative phosphorylation	YS vs. WS LYS vs. WS
*sev*	105230340	4	NC_064306.1 (93256–105304)	MAPK signaling pathway	LYS vs. WS
*LOC105231837*	105231837	2	NC_064304.1 (32268170–32288201)	Jak-STAT signaling pathway	YS vs. WS YS vs. LYS
*LOC105231839*	105231839	2	NC_064304.1 (32236821–32264955)	Jak-STAT signaling pathway	YS vs. WS YS vs. LYS
*LOC105231827*	105231827	2	NC_064304.1 (32354094–32363461)	Hippo signaling pathway	YS vs. WS YS vs. LYS
*Aldh7A1*	105232622	5	NC_064307.1 (4433597–4436786)	Valine, Leucine and Isoleucine degradation; Lysine degradation	YS vs. WS LYS vs. WS YS vs. LYS
*LOC105232613*	105232613	2	NC_064304.1 (402079–435348)	Glycerophospholipid metabolism	LYS vs. WS
*LOC105232615*	105232615	2	NC_064304.1 (467110–483000)	Porphyrin and chlorophyll metabolism	YS vs. LYS
*Prp19*	105229955	3	NC_064305.1 (79711816–79716434)	Ubiquitin-mediated proteolysis	YS vs. LYS
*Fancl*	105231847	2	NC_064304.1 (32106230–32107938)	Ubiquitin-mediated proteolysis	YS vs. LYS
*Ube4B*	105222370	1	NC_064303.1 (107841509–107851668)	Ubiquitin-mediated proteolysis	YS vs. LYS
*Nedd4*	105226493	5	NC_064307.1 (4497638–4502273)	Ubiquitin-mediated proteolysis	YS vs. LYS
*Herc4*	105226491	5	NC_064307.1 (4508221–4513959)	Ubiquitin-mediated proteolysis	YS vs. LYS
*Mdr50*	105223845	3	NC_064305.1 (8667810–8682111)	ABC Transporters	YS vs. LYS
*Mdr65*	105226497	5	NC_064307.1 (4482003–4493223)	ABC Transporters	YS vs. LYS
*Acat2*	105232621	5	NC_064307.1 (4436978–4438999)	Terpenoid backbone biosynthesis	YS vs. LYS
*LOC105223844*	105223844	3	NC_064305.1 (8687891–8694379)	Terpenoid backbone biosynthesis	YS vs. LYS
*Dhdds*	105227096	Unplaced Scaffold	NW_026038081.1 (117795–118974)	Terpenoid backbone biosynthesis	YS vs. LYS
*LOC105228052*	105228052	3	NC_064305.1 (905942–908404)	Biosynthesis of secondary metabolites	YS vs. WS LYS vs. WS YS vs. LYS
*LOC105229681*	105229681	3	NC_064305.1 (87358196–87359090)	Metabolism of xenobiotics by cytochrome P450	YS vs. WS LYS vs. WS YS vs. LYS

## Data Availability

The data presented in this study are available on request from the corresponding author.

## Supplementary Materials

The following supporting information can be downloaded at https://www.mdpi.com/article/10.3390/insects16010076/s1. Table S1: Chromaticity value of yellow, light-yellow and white scutella. Table S2: Statistics of SNP detection results. Figure S1: Three types of color of *B. dorsalis* scutellum. Figure S2: Rearing *B. dorsalis* indoors and trapping adults in orchards.
