# Supplementary material for: Molecular and Biochemical Mechanisms of Scutellum Color Variation in Bactrocera dorsalis Adults (Diptera: Tephritidae)
Source: Insects. 2025 Jan 14;16(1):76. doi: 10.3390/insects16010076 (PMC11765850; doi:10.3390/insects16010076)
Supplement: Supplementary file 1 [file insects-16-00076-s001.zip › insects-3380975-supplementary.pdf]

Supporting information

Table S1. Chromaticity value of yellow, light yellow and white scutella.

Color channel	Samples	YS	LYS	WS
Red intensity value	8	205.13 ± 4.38	208.50 ± 3.61	242.38 ± 5.22
Green intensity value	8	201.50 ± 4.32	195.25 ± 4.82	232.13 ± 10.99
Blue intensity value	8	10.13 ± 3.29	177.00 ± 8.63	233.13 ± 7.37

Table S2. Statistics of SNP detection results.

Category	Upstream	Missense	Exonic regions					Intronic	Splicing	Downstream	Upstream/ Downstream	Intergenic	ts	tv	Ts/tv	heterozygosity	Total
			Stop gain	Stop loss	Synonymous	Non-synonymous	Unknowns										
All	40544	0	0	0	66645	17580	0	761204	109	33711	4053	927023	1176971	735285	1.600	0	1912256
YS1	20203	0	0	0	36096	9095	0	373420	61	16607	2047	450932	578383	361416	1.600	2.140	939799
YS2	19926	0	0	0	35578	8911	0	364654	54	16228	1936	442311	567504	352701	1.609	1.985	920205
YS3	19883	0	0	0	35731	8911	0	361993	53	16297	1933	435591	561354	349471	1.606	2.064	910825
YS4	19991	0	0	0	35824	9123	0	366691	47	16540	1956	444670	570094	355569	1.603	2.081	925663
YS5	19801	0	0	0	36000	9112	0	368017	59	16246	2014	445926	571597	356687	1.602	2.073	927299
YS6	19944	0	0	0	36034	9112	0	368323	56	16389	2029	444343	571190	356109	1.603	2.155	944133
YS7	20225	0	0	0	36301	9246	0	374325	52	16643	2020	454017	581578	362555	1.604	2.075	927311
YS8	19992	0	0	0	35893	8965	0	367540	52	16244	2007	445856	571116	356195	1.603	2.068	926167
LYS1	19837	0	0	0	35529	8984	0	368186	58	16191	2025	444532	569865	356302	1.599	2.608	926167
LYS3	20143	0	0	0	36111	9099	0	372611	58	16663	2046	448569	576696	359415	1.604	2.116	936111
LYS4	19806	0	0	0	35929	9046	0	369573	47	16420	2078	442764	569709	355893	1.600	2.074	925602
LYS5	20275	0	0	0	36295	9255	0	374242	53	16621	1958	450202	578629	361739	1.599	2.138	940368
LYS6	20039	0	0	0	36080	9149	0	370908	49	16176	2040	450361	577122	358510	1.609	2.116	935632
LYS7	19968	0	0	0	35527	8998	0	365995	56	16225	2032	441145	566884	353737	1.602	2.036	920621
LYS8	22417	0	0	0	41572	10364	0	414212	62	18197	2231	500746	644908	399907	1.612	2.699	1044815
WS1	19961	0	0	0	35112	8986	0	367218	58	16549	2013	444755	570221	355206	1.605	2.051	925847
WS2	19219	0	0	0	34510	8796	0	353425	56	15721	1968	425878	547350	342215	1.599	1.882	889565
WS3	20065	0	0	0	35730	9108	0	370882	50	16526	1986	451575	577256	359735	1.604	2.117	936991
WS5	19545	0	0	0	34172	8837	0	360044	48	16077	1968	430815	555988	346944	1.602	1.935	902932
WS6	20148	0	0	0	35950	9107	0	370441	47	16551	1963	450453	576206	359303	1.603	2.115	935509
WS7	19243	0	0	0	34497	8702	0	353907	53	15837	1922	426001	547724	342233	1.60	1.903	889957
WS8	19791	0	0	0	35955	8995	0	362390	46	15948	1991	436654	562373	350004	1.606	1.994	912377

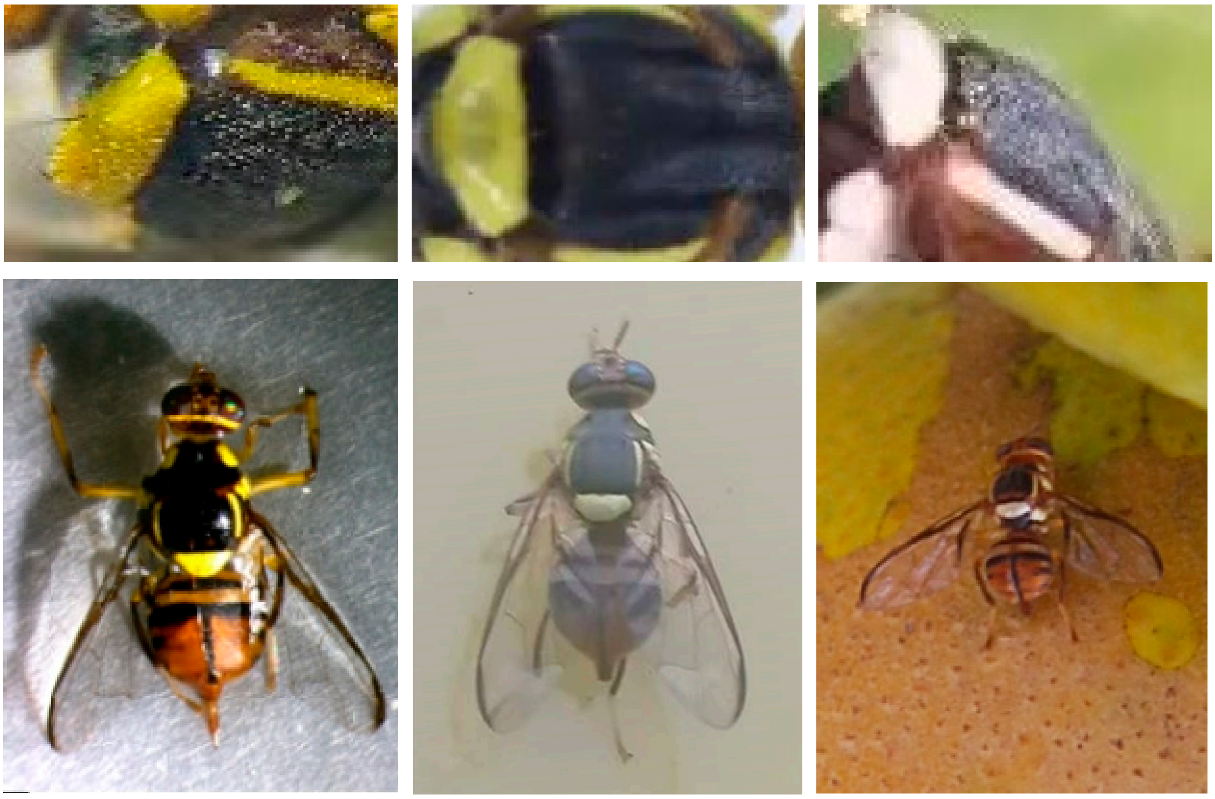

Figure S1. Three types of color of *B. dorsalis* scutellum. Left: yellow scutellum, Middle: light yellow scutellum, Right: white scutellum.

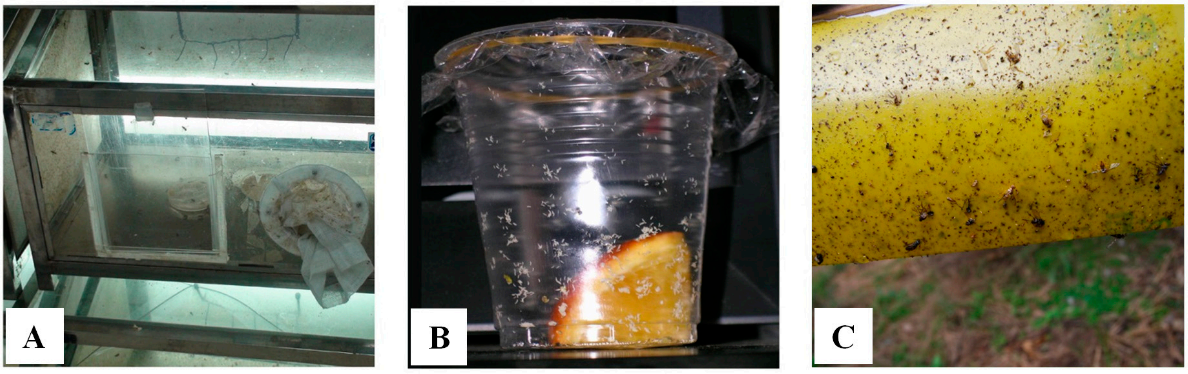

Figure S2. Rearing *B. dorsalis* in indoors and trapping adult in orchards. A: Rearing adults with different color scutella in rearing box; B: Oviposition cup wall with a large number of eggs; C: Trapping adults with yellow glue boards.
